# Neural Adaptive *H*_∞_ Sliding-Mode Control for Uncertain Nonlinear Systems with Disturbances Using Adaptive Dynamic Programming

**DOI:** 10.3390/e25121570

**Published:** 2023-11-22

**Authors:** Yuzhu Huang, Zhaoyan Zhang

**Affiliations:** College of Electronic and Information Engineering, Hebei University, Baoding 071002, China; zhangzhaoyan@hbu.edu.cn

**Keywords:** adaptive dynamic programming, disturbance observer, neural networks, optimal control, sliding-mode control

## Abstract

This paper focuses on a neural adaptive $H_{\infty }$ sliding-mode control scheme for a class of uncertain nonlinear systems subject to external disturbances by the aid of adaptive dynamic programming (ADP). First, by combining the neural network (NN) approximation method with a nonlinear disturbance observer, an enhanced observer framework is developed for estimating the system uncertainties and observing the external disturbances simultaneously. Then, based on the reliable estimations provided by the enhanced observer, an adaptive sliding-mode controller is meticulously designed, which can effectively counteract the effects of the system uncertainties and the separated matched disturbances, even in the absence of prior knowledge regarding their upper bounds. While the remaining unmatched disturbances are attenuated by means of $H_{\infty }$ control performance on the sliding surface. Moreover, a single critic network-based ADP algorithm is employed to learn the cost function related to the Hamilton–Jacobi–Isaacs equation, and thus, the $H_{\infty }$ optimal control is obtained. An updated law for the critic NN is proposed not only to make the Nash equilibrium achieved, but also to stabilize the sliding-mode dynamics without the need for an initial stabilizing control. In addition, we analyze the uniform ultimate boundedness stability of the resultant closed-loop system via Lyapunov’s method. Finally, the effectiveness of the proposed scheme is verified through simulations of a single-link robot arm and a power system.

## 1. Introduction

Within the last few decades, multifarious robust control design theories and methods have been proposed for uncertain nonlinear systems [1]. As one of the most efficient and widely used control methods, sliding-mode control (SMC) has garnered significant attention by reason of its simplicity, order reduction and inherent robustness against the matched uncertainties [2]. The classical SMC approach is to exert a discontinuous control to drive the system states onto a prescribed sliding manifold or surface [3]. As long as the sliding surface is reached, the system will become immune from the matched uncertainties and input disturbances. To remove the reaching phase, an integral SMC was developed by using the integral sliding manifold, including an integral term, which can enable the system states to reach and remain on the sliding manifold from the beginning [4,5,6]. Although towards a wide variety of actual systems, the relevant uncertainties and disturbances can be assumed to be matched in the design of control systems, there are also many physical systems, such as permanent magnet synchronous motors [7], underactuated aerial vehicles and robotic systems [8] directly affected by unmatched disturbances. Lately, several new approaches involving the integral SMC have been proposed to stabilize various systems with unmatched disturbances [9,10,11,12,13]. Among these methods, it is worth noticing that in [12,13], the impact of the separated unmatched disturbances would not be amplified after choosing a suitable projection matrix in a sliding manifold and were attenuated by the combination of the integral SMC with $H_{\infty }$ control theories. This provides a feasible and effective way to handle the unmatched disturbances and helps explore the relationships between integral SMC and $H_{\infty }$ control in nonlinear system control design.

In many instances, we expect the control policy not just to make the closed-loop system stable, but to possess certain optimality by minimizing the user-defined cost. For nonlinear systems, the settlement of associated optimal control problems requires solving the Hamilton–Jacobi–Bellman (HJB) equation. While considering $H_{\infty }$ optimal control, based on the dissipativity theory, it can be formulated as an $L_{2}$-gain control problem, which involves solving the Hamilton–Jacobi–Isaacs (HJI) equation [14]. However, the analytical solutions of both HJB and HJI equations are very hard or even impossible to obtain directly because of their inherent nonlinearities [15]. In recent years, a class of neural network (NN) and reinforcement learning (RL)-based intelligent optimization and control methods, referred to as adaptive dynamic programming (ADP), is becoming more and more striking and shows great application potential in solving various optimization problems, and effectively conquers the “curse of dimensionality” [15,16]. By now, many researchers have employed ADP to tackle a variety of optimal control problems for both discrete-time (DT) [17,18,19,20,21,22] and continuous-time (CT) systems [23,24,25,26,27,28]. Moreover, how to combine ADP with other robust methods to achieve better performance and stronger robustness for uncertain nonlinear systems is becoming a new research focus [29,30].

Recently, Modares et al. [31] proposed an online integral RL algorithm that incorporates a non-quadratic discounted cost function to address the constrained-input optimal tracking problem. Luo et al. [32] described an NN-based off-policy learning algorithm within the actor-critic framework to deal with the associated HJI equation, and this algorithm was later extended to find the near-optimal $H_{\infty }$ tracking control solution in [33]. Nevertheless, the influences of potential system or modeling uncertainties were not taken into account in the design. Wang et al. [34] introduced a robust neuro-optimal control approach for input-affine nonlinear systems with both matched and state-depended uncertainties. They achieved this by redesigning the cost function and selecting a suitable feedback gain, whereas the upper bound function of uncertainties is needed for redesigning the cost function to suppress these uncertainties. Mitra et al. [35] presented an optimal SMC scheme for the single-input cascade nonlinear systems with matched bounded disturbances. Fan et al. [36] investigated an adaptive actor–critic-based integral SMC strategy for CT nonlinear systems with unknown terms and input disturbances, where the initial stabilizing control requirement in the learning was quite stringent and limiting in practical applications. Qu et al. [37] developed an adaptive $H_{\infty }$ optimal SMC method in the presence of actuator faults and unmatched disturbances using the ADP algorithm, and further explored the optimal guaranteed cost SMC for constrained-input uncertain systems by formulating an auxiliary system and redefining the utility function [38]. Based on [37], combined with event-triggered mechanisms, Yang et al. [39] provided an event-triggered integral SMC design for nonlinear control-affine systems by leveraging the ADP technique. Note that these methods mentioned above rely on the availability of upper bounds for matched or unmatched disturbances, which may cause over-design and thus leads to an over-conservative control scheme. Additionally, in real-world scenarios, determining precise upper bounds of external disturbances is often a challenging task.

Inspired by the works mentioned earlier, we propose an adaptive neural $H_{\infty }$ SMC scheme for uncertain nonlinear systems subject to external disturbances using the ADP algorithm. Based on the enhanced observer system composed of the NN identifier and nonlinear disturbance observer (DO), an integral SMC is developed to counteract the impacts of the system uncertainties and the separated matched disturbances, as well as unknown approximation errors, without requiring prior knowledge of their upper bounds. While on the sliding manifold, the remaining unmatched disturbances are attenuated by $H_{\infty }$ optimal control solved by the single-network ADP algorithm. Moreover, the uniform ultimate boundedness stability of the resultant closed-loop system are guaranteed via the Lyapunov approach.

The principal contributions of this study can be enumerated as follows. First, unlike other existing schemes [34,35,36,37,38,39], based on the enhanced observer system, the proposed approach makes the designed sliding-mode controller independent from the relevant upper bounds of uncertainties and disturbances, which renders the implementation much easier and more practical and removes the assumption that the upper bounds need to be known in advance. Second, compared with the algorithms presented in [36,37], our approach can deal with both unknown nonlinear terms and unmatched external disturbances, where the single-network ADP is utilized to approximate an $H_{\infty }$ optimal control. Unlike typical actor–critic–disturbance network architectures, the single critic network structure may bring a simpler implementation, lower calculation amount, and avoid the numerical approximate errors arising from actor and disturbance networks. Third, we introduce an updated law for the critic NN, which not only achieves the Nash equilibrium, but also ensures the stability of the sliding-mode dynamics without the need for an initial stabilizing control in the learning.

The remainder of this paper is arranged as follows. Section 2 outlines the problem formulation and provides some necessary preliminaries. Section 3 describes the design of an integral SMC based on the enhanced observer system. Section 4 presents the application of the single-network ADP to obtain $H_{\infty }$ optimal control for the sliding-mode dynamics, along with stability analysis. Simulations of the robotic arm and a power system are given in Section 5, followed by a summary of this study in Section 6.

## 2. Problem Formulation

Consider the following uncertain perturbed nonlinear system as

(1) $$\dot{x}=f\left(x\right)+\Delta f\left(x\right)+\left(g(x)+\Delta g(x)\right)u+d,$$

where the state vector $x\in R^{n}$ is measurable, $u\in R^{m}$ is the control input, $f\left(x\right)\in R^{n}$ and $g\left(x\right)\in R^{n\times m}$ are the known system drift and input dynamics, respectively; Δf(x) and Δg(x) denote uncertain nonlinear terms that refer to either the inherent characteristics of the system or modeling uncertainties, while $d\in R^{n}$ represents the unknown external disturbances. Moreover, it is assumed that the system uncertainties Δf(x) and Δg(x) satisfy the matched condition, i.e., Δf(x)+Δg(x)u=g(x)w(x,u), then the system (1) is rewritten in the form of

(2) $$\dot{x}=f\left(x\right)+g\left(x\right)u+g\left(x\right)w\left(x,u\right)+d$$

with w(x,u) being the bounded lumped uncertain term. Let $\Omega \subseteq R^{n}$ be a compact set, and suppose that f(x)+g(x)u is Lipschitz continuous over Ω with f(0)=0. Besides, $d\in L_{2}\left[0,\infty \right]$ and its derivative $\dot{d}$ is bounded such that $∥\dot{d}∥\leq d_{M}$ with $d_{M}>0$. To avoid any confusion, ∥·∥ denotes the 2-norm of a vector or the Frobenius norm of a matrix hereafter, unless otherwise specified.

**Assumption** **1.** *The input matrix g(x) has a full column rank and is norm bounded with $g_{M}>0$, that is, $∥g\left(x\right)∥\leq g_{M}$ for any x. Moreover, the resulting left pseudoinverse $g^{+}\left(x\right)\in R^{m\times n}$ is given by $g^{+}\left(x\right)={\left(g^{T}\left(x\right)g\left(x\right)\right)}^{-1}g^{T}\left(x\right)$, which is bounded by $∥g^{+}\left(x\right)∥\leq b_{M}$, where $b_{M}$, $g_{M}$ are known positive constants.*

Based on Assumption 1, *d* is then decomposed into the matched and unmatched components through the projection of *d* onto the input matrix g(x) as

(3) $$d=g\left(x\right)g^{+}\left(x\right)d+\left(I-g\left(x\right)g^{+}\left(x\right)\right)d,$$

where *I* denotes an identity matrix of appropriate dimensions, and $g^{+}\left(x\right)$ is the left pseudoinverse of g(x). It should be noted that Assumption 1 is somewhat restrictive, which may lessen the applicability scope of the proposed approach to some extent. However, many real-world physical systems, such as the satellite dynamics, the hypersonic flight vehicle and overhead crane systems, have such a property to make this assumption valid [15,20].

To deal with the uncertain nonlinear system (1) with external disturbances, an enhanced observer system is first constructed for estimating the uncertain terms and observing the unknown disturbances simultaneously. Then, based on the reliable estimations, an integral SMC is developed to counteract the impacts of the system uncertainties and the separated matched disturbances, as well as unknown approximation errors, without requiring prior knowledge of their upper bounds. Meanwhile, the remaining unmatched disturbances are attenuated by $H_{\infty }$ optimal control on the sliding surface. Moreover, the single-network ADP algorithm is employed to learn the cost function related to the Hamilton–Jacobi–Isaacs equation, and then, the $H_{\infty }$ optimal control is obtained. What is more, a weight updating law is formulated to ensure both the achievement of Nash equilibrium and the stabilization of sliding-mode dynamics during the learning process.

## 3. Integral SMC Design Based on the Enhanced Observer System

Recalling the NN universal approximation property, the uncertain term w(x,u) can be represented by a three-layered NN as

(4) $$w\left(x,u\right)=W_{o}^{T}\sigma \left(V_{o}^{T}\bar{x}\right)+\varepsilon_{o}\left(x\right),$$

where $W_{o}\in R^{l_{o}\times m}$ and $V_{o}\in R^{\left(n+m\right)\times l_{o}}$ denote unknown ideal weight matrices between the output and hidden, and hidden and input layers, respectively; $\bar{x}={\left[x^{T},u^{T}\right]}^{T}\in R^{n+m}$ is the NN input, $\sigma \left(\cdot \right)\in R^{l_{o}}$ represents the activation function with $l_{o}$ hidden layer neurons, and $\varepsilon_{o}\left(x\right)\in R^{m}$ stands for the NN reconstruction error. To simplify the learning process, only the weights of $W_{o}$ are adapted online, while $V_{o}$ is an initialized set with random values and then remains unchanged during the weight updating process [16].

The NN identifier is designed by

(5) $$\dot{\hat{x}}=A\hat{x}+f\left(x\right)-Ax+g\left(x\right)u+g\left(x\right){\hat{W}}_{o}^{T}\sigma \left(z\right)+d,$$

where *A* is a Hurwitz matrix, $\hat{x}$ is the identifier state, ${\hat{W}}_{o}$ is the estimate of $W_{o}$, and the activation function $\sigma \left(z\right)=\sigma \left(V_{o}^{T}\bar{x}\right)$ with $z=V_{o}^{T}\bar{x}$. Since the unknown disturbance term *d* is needed in (5), inspired by [11], a nonlinear DO is introduced for obtaining $\hat{d}$, namely, the estimated value of *d*.

Then, combining the NN identifier with a nonlinear DO, an enhanced observer system is constructed as

(6) $$\begin{array}{cc} \dot{\hat{x}}= & A\hat{x}+f\left(x\right)-Ax+g\left(x\right)u+g\left(x\right){\hat{W}}_{o}^{T}\sigma \left(z\right)+\hat{d}\\ \dot{d_{0}}= & -l\left(x\right)(f\left(x\right)+g\left(x\right)u+g\left(x\right){\hat{W}}_{o}^{T}\sigma \left(z\right)+d_{0}+p\left(x\right)), \end{array}$$

with $\hat{d}=d_{0}+p\left(x\right)$, where $d_{0}$ is an auxiliary variable, and p(x) is a designed state-dependent function and brings out the gain function l(x) such that $l\left(x\right)={\left(\partial p\left(x\right)/\partial x\right)}^{T}$. Following (6), we have

(7) $$\dot{\hat{d}}=-l\left(x\right)\hat{d}+l\left(x\right)d+l\left(x\right)g\left(x\right){\tilde{W}}_{o}^{T}\sigma \left(z\right)+l\left(x\right)g\left(x\right)\varepsilon_{o}\left(x\right),$$

where ${\tilde{W}}_{o}=W_{o}-{\hat{W}}_{o}$ represents the NN weight estimation error. Let $\tilde{x}=x-\hat{x}$ and $\tilde{d}=d-\hat{d}$ be the state and disturbance estimation errors, respectively. Subtracting (5) from (2) and combining with (7), we obtain the coupled error dynamics of (6) as follows:

(8) $$\begin{array}{cc} \dot{\tilde{x}} & =A\tilde{x}+g\left(x\right){\tilde{W}}_{o}^{T}\sigma \left(z\right)+\tilde{d}+g\left(x\right)\varepsilon_{o}\left(x\right),\\ \dot{\tilde{d}} & =-l\left(x\right)\tilde{d}-l\left(x\right)g\left(x\right){\tilde{W}}_{o}^{T}\sigma \left(z\right)+\dot{d}-l\left(x\right)g\left(x\right)\varepsilon_{o}\left(x\right). \end{array}$$

Before proceeding, we introduce a common assumption for stability analysis [15,16].

**Assumption** **2.** *For the identifier NN, there are known positive constants $\sigma_{M}$, $\varepsilon_{M}$, $W_{M}$ and $V_{M}$ in the sense that $∥\sigma \left(z\right)∥\leq \sigma_{M}$, $∥\varepsilon_{o}\left(x\right)∥\leq \varepsilon_{M}$, $∥W_{o}∥\leq W_{M}$ and $∥V_{o}∥\leq V_{M}$, respectively.*

**Lemma** **1.** *Considering the system (2) and the coupled error dynamics (8), let the identifier NN weight ${\hat{W}}_{o}$ be updated by*

(9) $${\dot{\hat{W}}}_{o}=-\eta_{1}\sigma \left(z\right){\tilde{x}}^{T}A^{-1}g\left(x\right)-\eta_{2}(∥\tilde{x}∥+1){\hat{W}}_{o},$$

*where $\eta_{1}$, $\eta_{2}$ are the positive updating ratios. Moreover, we select parameter matrices A, P and gain function l(x) to satisfy*

(10) $$P^{T}P-l\left(x\right)-l^{T}\left(x\right)+l\left(x\right)g\left(x\right)g^{T}\left(x\right)l^{T}\left(x\right)\leq -\rho I$$

*with ρ>0. Then all the estimation errors $\tilde{x}$, $\tilde{d}$, and ${\tilde{W}}_{0}$ are uniformly ultimately bounded (UUB).*

**Proof.**  Consider the Lyapunov function candidate given by

(11) $$L_{1}=\frac{1}{2}{\tilde{x}}^{T}P\tilde{x}+\frac{1}{2}{\tilde{d}}^{T}\tilde{d}+\frac{1}{2}\mathrm{tr}\left\{{\tilde{W}}_{o}^{T}{\tilde{W}}_{o}\right\},$$

where $L_{11}={\tilde{x}}^{T}P\tilde{x}/2+{\tilde{d}}^{T}\tilde{d}/2$, $L_{12}=\mathrm{tr}\left\{{\tilde{W}}_{o}^{T}{\tilde{W}}_{o}\right\}/2$, and $P=P^{T}$ is positive definite, which together with some matrix Λ>0 satisfies $A^{T}P+PA=-\Lambda$ for the Hurwitz matrix *A*. By taking the time derivative of $L_{11}$ and substituting the coupled error dynamics (8), we can obtain

(12) $$\begin{array}{cc} {\dot{L}}_{11}= & \frac{1}{2}{\tilde{x}}^{T}\left(A^{T}P+PA\right)\tilde{x}+{\tilde{x}}^{T}P\tilde{d}+{\tilde{x}}^{T}Pg\left(x\right)\varepsilon_{o}\left(x\right)+{\tilde{x}}^{T}Pg\left(x\right){\tilde{W}}_{o}^{T}\sigma \left(z\right)-{\tilde{d}}^{T}l\left(x\right)\\ \; & \times g\left(x\right){\tilde{W}}_{o}^{T}\sigma \left(z\right)-\frac{1}{2}{\tilde{d}}^{T}\left(l\left(x\right)+l^{T}\left(x\right)\right)\tilde{d}-{\tilde{d}}^{T}l\left(x\right)g\left(x\right)\varepsilon_{o}\left(x\right)+{\tilde{d}}^{T}\dot{d}. \end{array}$$

Based on Assumption 2, together with Young’s inequality, it follows:

(13) $$\begin{array}{cc} {\dot{L}}_{11}\leq & -\frac{1}{2}{\tilde{x}}^{T}\Lambda \tilde{x}+\frac{1}{2}{\tilde{x}}^{T}\tilde{x}+\frac{1}{2}{\tilde{d}}^{T}\left(P^{T}P-l\left(x\right)-l^{T}\left(x\right)+l\left(x\right)g\left(x\right)g^{T}\left(x\right)l^{T}\left(x\right)\right)\tilde{d}\\ \; & +{\tilde{d}}^{T}\dot{d}+{\tilde{x}}^{T}Pg\left(x\right){\tilde{W}}_{o}^{T}\sigma \left(z\right)+{\tilde{x}}^{T}Pg\left(x\right)\varepsilon_{o}\left(x\right)+\sigma_{M}^{2}{∥{\tilde{W}}_{o}∥}^{2}+\varepsilon_{M}^{2}. \end{array}$$

Considering (10), (13) is rewritten as

(14) $$\begin{array}{cc} {\dot{L}}_{11}\leq & -\frac{1}{2}\tau {\tilde{x}}^{T}\tilde{x}-\frac{1}{2}\rho {\tilde{d}}^{T}\tilde{d}+{\tilde{x}}^{T}Pg\left(x\right){\tilde{W}}_{o}^{T}\sigma \left(z\right)+{\tilde{x}}^{T}Pg\left(x\right)\varepsilon_{o}\left(x\right)\\ \; & +{\tilde{d}}^{T}\dot{d}+\sigma_{M}^{2}∥{\tilde{W}}_{o}{∥}^{2}+\varepsilon_{M}^{2}, \end{array}$$

where $\tau =\lambda_{\min}\left(\Lambda \right)-1>0$ ensured by properly selecting positive definite matrix Λ and its minimum eigenvalue $\lambda_{\min}\left(\Lambda \right)$.

Combining with (9), ${\dot{L}}_{12}$ is derived as

(15) $$\begin{array}{c} {\dot{L}}_{12}=\mathrm{tr}\{\eta_{1}{\tilde{W}}_{o}^{T}\sigma \left(z\right){\tilde{x}}^{T}A^{-1}g\left(x\right)+\eta_{2}{\tilde{W}}_{o}^{T}∥\tilde{x}∥{\hat{W}}_{o}+\eta_{2}{\tilde{W}}_{o}^{T}{\hat{W}}_{o}\}. \end{array}$$

With the inequality $\mathrm{tr}\{{\tilde{W}}_{o}^{T}{\hat{W}}_{o}\}\leq ∥W_{o}{∥}^{2}/2-∥{\tilde{W}}_{o}{∥}^{2}/2$, (15) becomes

$$\begin{array}{c} {\dot{L}}_{12}\leq \mathrm{tr}\{\eta_{1}{\tilde{W}}_{o}^{T}\sigma \left(z\right){\tilde{x}}^{T}A^{-1}g\left(x\right)\}+\mathrm{tr}\{\eta_{2}{\tilde{W}}_{o}^{T}∥\tilde{x}∥{\hat{W}}_{o}\}+\frac{\eta_{2}}{2}∥W_{o}{∥}^{2}-\frac{\eta_{2}}{2}{∥{\tilde{W}}_{o}∥}^{2}. \end{array}$$

Note that the relationship $\mathrm{tr}\left\{A^{T}B\right\}=B^{T}A$ for all $A\in R^{n}$, $B\in R^{n}$ and the inequality $\mathrm{tr}\left\{{\tilde{W}}_{o}^{T}\left(W_{o}-{\tilde{W}}_{o}\right)\right\}\leq W_{M}∥{\tilde{W}}_{o}∥-∥{\tilde{W}}_{o}{∥}^{2}$, we can have

(16) $$\begin{array}{cc} {\dot{L}}_{12}\leq & \eta_{1}\sigma_{M}g_{M}∥\tilde{x}∥∥A^{-1}∥∥{\tilde{W}}_{o}∥+\eta_{2}W_{M}∥\tilde{x}∥∥{\tilde{W}}_{o}∥-\eta_{2}∥\tilde{x}∥∥{\tilde{W}}_{o}{∥}^{2}+\frac{\eta_{2}}{2}{∥W_{o}∥}^{2}\\ \; & -\frac{\eta_{2}}{2}{∥{\tilde{W}}_{o}∥}^{2}. \end{array}$$

By combining (14) and (16) and taking their norms, one can derive an upper bound for ${\dot{L}}_{1}\left(t\right)$ as

(17) $$\begin{array}{cc} {\dot{L}}_{1}\leq & -\frac{1}{2}\tau ∥\tilde{x}{∥}^{2}+(g_{M}\varepsilon_{M}∥P∥+\left(g_{M}\sigma_{M}∥P∥+\eta_{1}g_{M}\sigma_{M}∥A^{-1}∥+\eta_{2}W_{M}\right)∥{\tilde{W}}_{o}∥-\eta_{2}\\ \; & \times ∥{\tilde{W}}_{o}{∥}^{2})∥\tilde{x}∥-\frac{1}{2}\rho ∥\tilde{d}{∥}^{2}+d_{M}∥\tilde{d}∥+\frac{\eta_{2}}{2}∥W_{o}{∥}^{2}-\frac{\eta_{2}-2\sigma_{M}^{2}}{2}{∥{\tilde{W}}_{o}∥}^{2}+\varepsilon_{M}^{2}. \end{array}$$

Select $\eta_{2}\geq 2\sigma_{M}^{2}$ and complete the square with respect to $∥{\tilde{W}}_{o}∥$, then (17) becomes

(18) $$\begin{array}{cc} {\dot{L}}_{1}\leq & -\frac{1}{2}\tau ∥\tilde{x}{∥}^{2}-\frac{1}{2}\rho ∥\tilde{d}{∥}^{2}+\left(g_{M}\varepsilon_{M}∥P∥-\eta_{2}{\left(∥{\tilde{W}}_{o}∥-\Theta_{1}\right)}^{2}+\eta_{2}{∥\Theta_{1}∥}^{2}\right)∥\tilde{x}∥\\ \; & +d_{M}∥\tilde{d}\left(t\right)∥+\Theta_{2}, \end{array}$$

where

$$\begin{array}{c} \Theta_{1}=\frac{g_{M}\sigma_{M}∥P∥+\eta_{1}g_{M}\sigma_{M}∥A^{-1}∥+\eta_{2}W_{M}}{2\eta_{2}},\;\;\;\;\;\Theta_{2}=\frac{\eta_{2}{∥W_{o}∥}^{2}+2\varepsilon_{M}^{2}}{2}. \end{array}$$

Define

$$e_{xd}=\left[\begin{array}{c} \tilde{x}\\ \tilde{d} \end{array}\right],\;\;\;\;\;\;\;\;\;E_{o}=\frac{1}{2}\left[\begin{array}{c} \tau I\;\;\;\;0\\ 0\;\;\;\;\rho I \end{array}\right]$$

and $B_{o}=\left[g_{M}\varepsilon_{M}∥P∥+\eta_{2}{∥\Theta_{1}∥}^{2},d_{M}\right]$, we can further derive

(19) $${\dot{L}}_{1}\leq -\lambda_{\min}\left(E_{o}\right)∥e_{xd}{∥}^{2}+∥B_{o}∥∥e_{xd}∥+\Theta_{2}.$$

Therefore, we can conclude that ${\dot{L}}_{1}<0$ only if $∥e_{xd}\left(t\right)∥$ satisfies

$$∥e_{xd}∥>\frac{∥B_{o}∥}{2\lambda_{\min}\left(E_{o}\right)}+\sqrt{\frac{∥B_{o}{∥}^{2}}{4\lambda_{\min}^{2}\left(E_{o}\right)}+\frac{\Theta_{2}}{\lambda_{\min}\left(E_{o}\right)}}.$$

Furthermore, according to the Lyapunov extension theorem [16], when the inequality (10) holds by selecting proper matrices, we can infer that all the estimation errors $\tilde{x}$, $\tilde{d}$, and ${\tilde{W}}_{o}$ are UUB. □

**Remark** **1.** *The gain function matrix l(x) is an important design parameter that can be chosen as linear or nonlinear functions. When the form of system function g(x) is simple, it can be easy to find the function l(x) that satisfies the inequality (10) by substituting appropriate functions into (10). However, if the form of system function g(x) is complex, the trial and error method is employed to select appropriate function l(x) that meets the inequality (10). Although there is no universal design procedure for designing l(x), experience has shown that it is not difficult to find a suitable l(x) for specific applications [36,37].*

To effectively handle both system uncertainties and external disturbances, we propose a compound $H_{\infty }$ optimal SMC scheme that combines the integral SMC with $H_{\infty }$ control theories. This compound controller is formulated as

(20) $$u=u_{d}+u_{c},$$

where $u_{d}$ represents the discontinuous control designed to steer the system trajectories towards and maintain them on the sliding surface, thereby eliminating the effects of matched uncertainties and disturbances. $u_{c}$ denotes the continuous control derived to guarantee the system stability and achieve near-optimal performance under the remaining unmatched disturbances on sliding surfaces.

Accordingly, we define the integral sliding surface as follows:

(21) $$s\left(x\right)=S_{0}\left(x\right)-S_{0}\left(x_{0}\right)-\int_{0}^{t}G\left(x\right)\left(f\left(x\right)+g\left(x\right)u_{c}\right)dv,$$

where $x_{0}$ denotes the initial state, $S_{0}\left(x\right)\in R^{m}$ and $G\left(x\right)=\partial S_{0}\left(x\right)/\partial x\in R^{m\times n}$. Moreover, it follows from Assumption 1 that a suitable matrix G(x) can be found such that the product G(x)g(x) is invertible.

Taking the time derivative of s(x) as

(22) $$\dot{s}\left(x\right)=G\left(x\right)\left(g\left(x\right)u_{d}+g\left(x\right)w\left(x,u\right)+d\right).$$

By incorporating the valid estimators $\hat{d}$ and ${\hat{W}}_{o}$, $u_{d}$ is devised as

(23) $$\begin{array}{cc} u_{d}= & -{\left(G\left(x\right)g\left(x\right)\right)}^{-1}\left(G\left(x\right)\hat{d}+G\left(x\right)g\left(x\right){\hat{W}}_{o}^{T}\sigma \left(z\right)+\mu \mathrm{sgn}\left(s\right)+\frac{G\left(x\right)G^{T}\left(x\right)s}{∥s^{T}G\left(x\right)∥}\zeta \right), \end{array}$$

where μ>0, $\mathrm{sgn}\left(s\right)\in R^{m}$ is the sign function, and ζ∈R is generated by

(24) $$\dot{\zeta }=\kappa ∥s^{T}G\left(x\right)∥$$

with κ>0. In particular, it is noted that ζ is designed to tackle the unknown bounds of the approximation errors arisen from the estimated terms $\hat{d}$ and ${\hat{W}}_{o}$.

Considering the specific implementation of $\hat{d}$ and ${\hat{W}}_{o}$ in (23), we define $\zeta_{e}=\tilde{d}+g\left(x\right){\tilde{W}}_{o}\sigma \left(z\right)+g\left(x\right)\varepsilon_{o}\left(x\right)$ to represent the approximation errors. Based on the previous analysis and the boundedness of g(x), $\zeta_{e}$ is bounded as $∥\zeta_{e}∥\leq \zeta_{M}$ for an unknown positive constant $\zeta_{M}$. To estimate $\zeta_{M}$, we design ζ as defined in (24), and the estimation error is calculated as $\tilde{\zeta }=\zeta_{M}-\zeta$.

**Theorem** **1.** *Considering system (2) with the sliding surface (21), the discontinuous control $u_{d}$ is devised by (23) with the adaptive law (24), then it can guarantee the convergence of the sliding surface s to zero from the beginning.*

**Proof.**  Choose the positive definite Lyapunov function candidate as

$$L_{s}=\frac{1}{2}s^{T}s+\frac{1}{2}\kappa^{-1}{\tilde{\zeta }}^{2}.$$

Along with the system (2), $\dot{L_{s}}\left(t\right)$ is derived as

(25) $$\begin{array}{cc} \dot{L_{s}} & =s^{T}\dot{s}-\kappa^{-1}\tilde{\zeta }\dot{\zeta }\\ \; & =s^{T}G\left(x\right)\left(g\left(x\right)u_{d}+g\left(x\right)w\left(x,u\right)+d\right)-\kappa^{-1}\tilde{\zeta }\dot{\zeta }. \end{array}$$

Substituting (23) and (24) into (25), we can have

$$\begin{array}{cc} \dot{L_{s}} & =s^{T}G\left(x\right)(\tilde{d}+g\left(x\right){\tilde{W}}_{o}^{T}\sigma \left(z\right)+g\left(x\right)\varepsilon_{o}\left(x\right))-\mu s^{T}\mathrm{sgn}\left(s\right)-∥s^{T}G\left(x\right)∥\zeta -\tilde{\zeta }∥s^{T}G\left(x\right)∥\\ \; & =s^{T}G\left(x\right)\zeta_{e}-\mu s^{T}\mathrm{sgn}\left(s\right)-∥s^{T}G\left(x\right)∥\zeta -\tilde{\zeta }∥s^{T}G\left(x\right)∥. \end{array}$$

Using $∥\zeta_{e}∥\leq \zeta_{M}$ and the estimation error $\tilde{\zeta }=\zeta_{M}-\zeta$ yields

(26) $$\begin{array}{cc} \dot{L_{s}} & \leq ∥s^{T}G\left(x\right)∥\zeta_{M}-∥s^{T}G\left(x\right)∥\zeta -\tilde{\zeta }∥s^{T}G\left(x\right)∥-\mu s^{T}\mathrm{sgn}\left(s\right)\\ \; & \leq -\mu s^{T}\mathrm{sgn}\left(s\right). \end{array}$$

Thus, it is shown from (26) that $\dot{L_{s}}\leq -\mu {∥s∥}_{1}<0$ for any s≠0, where ${∥s∥}_{1}$ denotes the vector 1-norm. This means the asymptotic stability and convergence of sliding mode motion s(x)=0 can be guaranteed. Moreover, according to (21), the sliding surface $s(x_{0})=0$ when t=0, which implies that the system states start on the sliding surface, thus avoiding the need for a separate reaching phase. □

From Theorem 1, it is clear that the stable sliding motion s(x)=0 exists from the initial time; that is, for all t≥0, s(x)=0 and $\dot{s}\left(x\right)=0$. Moreover, the equivalent control method is utilized to obtain the sliding-mode dynamics. Combining $\dot{s}\left(x\right)=0$ with (3) and (22), the equivalent control can be derived as

(27) $$u_{\mathrm{deq}}=-{\left(G\left(x\right)g\left(x\right)\right)}^{-1}G\left(x\right)\left(I-g\left(x\right)g^{+}\left(x\right)\right)d-g^{+}\left(x\right)d-w\left(x,u\right).$$

Then, substitute $u_{\mathrm{deq}}$ into (2), the sliding-mode dynamics without matched uncertain term and disturbance component is

(28) $$\dot{x}=f\left(x\right)+g\left(x\right)u_{c}+\Gamma \left(x\right)d_{u},$$

where $\Gamma \left(x\right)=I-g\left(x\right){\left(G\left(x\right)g\left(x\right)\right)}^{-1}G\left(x\right)$, $d_{u}=\left(I-g\left(x\right)g^{+}\left(x\right)\right)d$ is the unmatched component of the external disturbance in (3). In order to reduce the influence of multiplier matrix Γ(x) and minimize the unmatched disturbance $\Gamma \left(x\right)d_{u}$, an optimal projection matrix $G^{*}\left(x\right)$ within Γ(x) is provided in the following Lemma.

**Lemma** **2.** Considering nonlinear system (2) with Assumption 1, the optimal projection matrix $G^{*}\left(x\right)$ is selected as $G^{*}\left(x\right)=g^{+}\left(x\right)$, which not only minimizes the norm $∥\Gamma \left(x\right)d_{u}∥$, but also makes the relation $\Gamma \left(x\right)d_{u}=d_{u}$ hold.

**Proof.**  The proof can refer to Theorem 1 in [12]. □

As a result, with the relation $\Gamma \left(x\right)d_{u}=d_{u}$, we can express (28) as

(29) $$\dot{x}=f\left(x\right)+g\left(x\right)u_{c}+d_{u},$$

which means that the discontinuous control $u_{d}$ in (23) can fully counteract the impacts of the matched uncertainties and disturbances.

Notice that in (20), $u_{c}$ aims not only to suppress the remaining unmatched disturbances on sliding surface, but also to achieve a near-optimal performance for sliding-mode dynamics (29). This formulation can be seen as a nonlinear $H_{\infty }$ optimal control problem, which is known to be challenging to solve directly. In the following, we will demonstrate how to find an approximate $H_{\infty }$ optimal control solution by using the single-network ADP algorithm.

## 4. *H*_∞_ Control Design for Sliding-Mode Dynamics

Considering (3) and (29), the sliding-mode dynamics is represented as

(30) $$\dot{x}=f\left(x\right)+g\left(x\right)u_{c}+k\left(x\right)d,$$

with $k\left(x\right)=I-g\left(x\right)g^{+}\left(x\right)$. Since g(x) and $g^{+}\left(x\right)$ are bounded, it follows that the function k(x) is also bounded by $∥k\left(x\right)∥\leq k_{M}$ with $k_{M}>0$.

For attenuating the remaining unmatched disturbances k(x)d, the corresponding $H_{\infty }$ control problem of sliding-mode dynamics is established, which aims to seek a feedback control $u_{c}$ to stabilize the system and achieve $L_{2}$-gain no larger than γ, that is,

(31) $$\int_{0}^{\infty }\left(x^{T}Qx+u_{c}^{T}Ru_{c}\right)dv\leq \gamma^{2}\int_{0}^{\infty }d^{T}d\;dv,$$

where *Q* and *R* are positive definite matrices with appropriate dimensions, and γ>0 refers to the level of the disturbance attenuation. Based on [32,33], by treating the disturbance *d* as the other system input, we can reframe the $H_{\infty }$ optimal control problem for system (30) as a two-player zero-sum game with the following infinite-horizon cost function:

(32) $$V\left(x\right)=\int_{t}^{\infty }\left(x^{T}Qx+u_{c}^{T}Ru_{c}-\gamma^{2}d^{T}d\right)dv.$$

Assuming that $V\left(x\right)\in C^{1}$, the Hamiltonian function with the associated admissible control pair $\left(u_{c},d\right)$ is defined as

(33) $$H\left(x,\nabla V,u_{c},d\right)=x^{T}Qx+u_{c}^{T}Ru_{c}-\gamma^{2}d^{T}d+{\left(\nabla V\right)}^{T}\left(f\left(x\right)+g\left(x\right)u_{c}+k\left(x\right)d\right)$$

with ∇V=∂V(x)/∂x. From Bellman’s optimality principle, it follows that the optimal cost function $V^{*}\left(x\right)$ satisfies the HJI equation

(34) $$0=\min_{u_{c}}\max_{d}H\left(x,\nabla V^{*},u_{c},d\right)$$

with $\nabla V^{*}=\partial V^{*}\left(x\right)/\partial x$. Moreover, according to the zero-sum game theory [16], we have the following Nash condition

(35) $$\min_{u_{c}}\max_{d}H\left(x,\nabla V^{*},u_{c},d\right)=\max_{d}\min_{u_{c}}H\left(x,\nabla V^{*},u_{c},d\right),$$

which ensures the existence of saddle point $\left(u_{c}^{*},d^{*}\right)$ of the HJI Equation (34). Then, applying the stationary condition, one can derive the optimal control $u_{c}^{*}$ and worst disturbance $d^{*}$ as

(36) $$\begin{array}{cc} u_{c}^{*} & =-\frac{1}{2}R^{-1}g^{T}\left(x\right)\nabla V^{*}, \end{array}$$

(37) $$\begin{array}{cc} d^{*} & =\frac{1}{2\gamma^{2}}k^{T}\left(x\right)\nabla V^{*}. \end{array}$$

By substituting (36) and (37) into (33), the HJI equation associated with $\nabla V^{*}$ becomes

(38) $$\begin{array}{cc} 0= & x^{T}Qx+{\left(\nabla V^{*}\right)}^{T}f\left(x\right)-\frac{1}{4}{\left(\nabla V^{*}\right)}^{T}g\left(x\right)R^{-1}g^{T}\left(x\right)\nabla V^{*}\\ \; & +\frac{1}{4\gamma^{2}}{\left(\nabla V^{*}\right)}^{T}k^{T}\left(x\right)k\left(x\right)\nabla V^{*}. \end{array}$$

Due to the highly nonlinear nature of the relevant HJI equation, obtaining its analytical solution is extremely difficult, if not impossible. To overcome this challenge, we propose an online optimal algorithm that learns the solution of the HJI equation and achieves $H_{\infty }$ optimal control. This is accomplished through the use of single-network ADP, where only one critic network, implemented by NN, is adopted to approximate the cost function $V^{*}$ related to (38). Therefore, by using the critic NN with $l_{c}$ neurons, $V^{*}$ is represented over a set Ω as follows:

(39) $$V^{*}\left(x\right)=W_{c}^{T}\sigma_{c}\left(x\right)+\varepsilon_{c}\left(x\right)$$

with the ideal weight vector $W_{c}\in R^{l_{c}}$ being unknown, the vector of activation functions $\sigma_{c}\left(x\right)\in R^{l_{c}}$ and the reconstruction error $\varepsilon_{c}\left(x\right)$. Meanwhile, we have the gradient vector

(40) $$\nabla V^{*}={\left(\nabla \sigma_{c}\right)}^{T}W_{c}+\nabla \varepsilon_{c}$$

with $\nabla \sigma_{c}=\partial \sigma_{c}\left(x\right)/\partial x$ and $\nabla \varepsilon_{c}=\partial \varepsilon_{c}\left(x\right)/\partial x$.

By combining (36), (37) and (40), it is easy to get

(41) $$\begin{array}{cc} u_{c}^{*} & =-\frac{1}{2}R^{-1}g^{T}\left(x\right)\left({\left(\nabla \sigma_{c}\right)}^{T}W_{c}+\nabla \varepsilon_{c}\right), \end{array}$$

(42) $$\begin{array}{cc} d^{*} & =\frac{1}{2\gamma^{2}}k^{T}\left(x\right)\left({\left(\nabla \sigma_{c}\right)}^{T}W_{c}+\nabla \varepsilon_{c}\right). \end{array}$$

Substituting (41) and (42) into (33), the HJI equation becomes

(43) $$\begin{array}{cc} 0= & H(x,\nabla V^{*},u_{c}^{*},d^{*})\\ = & x^{T}Qx+W_{c}^{T}\nabla \sigma_{c}f\left(x\right)-\frac{1}{4}W_{c}^{T}\nabla \sigma_{c}D{\left(\nabla \sigma_{c}\right)}^{T}W_{c}-\varepsilon_{\mathrm{HJI}}, \end{array}$$

where $D=g\left(x\right)R^{-1}g^{T}\left(x\right)-k\left(x\right)k^{T}\left(x\right)/\gamma^{2}$, and the approximate error $\varepsilon_{\mathrm{HJI}}$ is defined as $\varepsilon_{\mathrm{HJI}}=-{\left(\nabla \varepsilon_{c}\right)}^{T}f\left(x\right)+W_{c}^{T}\nabla \sigma_{c}D\nabla \varepsilon_{c}/2+{\left(\nabla \varepsilon_{c}\right)}^{T}D\nabla \varepsilon_{c}/4$ due to the NN reconstruction error. Furthermore, taking into account $∥k\left(x\right)∥\leq k_{M}$ and $∥g\left(x\right)∥\leq g_{M}$, we can infer that there exists a positive constant $D_{M}$ in the sense that $∥D∥\leq D_{M}$.

Because $W_{c}$ in (39) is unknown, the critic NN with the estimated weights approximates the cost function in the form of

(44) $$\hat{V}\left(x\right)={\hat{W}}_{c}^{T}\sigma_{c}\left(x\right),$$

where ${\hat{W}}_{c}$ denotes the estimated values of $W_{c}$. In addition, we can obtain

(45) $$\nabla \hat{V}={\left(\nabla \sigma_{c}\right)}^{T}{\hat{W}}_{c}.$$

By using (36), (37) and (45), the approximate forms of (41) and (42) are derived as

(46) $$\begin{array}{cc} {\hat{u}}_{c} & =-\frac{1}{2}R^{-1}g^{T}\left(x\right){\left(\nabla \sigma_{c}\right)}^{T}{\hat{W}}_{c}, \end{array}$$

(47) $$\begin{array}{cc} {\hat{d}}_{w} & =\frac{1}{2\gamma^{2}}k^{T}\left(x\right){\left(\nabla \sigma_{c}\right)}^{T}{\hat{W}}_{c}. \end{array}$$

Then, incorporating (46) and (47) into (43), we have the approximate Hamiltonian as follows:

(48) $$\begin{array}{cc} H(x,{\hat{W}}_{c},{\hat{u}}_{c},{\hat{d}}_{w}) & =x^{T}Qx+{\hat{W}}_{c}^{T}\nabla \sigma_{c}f\left(x\right)-\frac{1}{4}{\hat{W}}_{c}^{T}\nabla \sigma_{c}D{\left(\nabla \sigma_{c}\right)}^{T}{\hat{W}}_{c}. \end{array}$$

Subtracting (43) from (48), the corresponding Hamiltonian error is defined as

$$\begin{array}{c} e_{c}=H\left(x,{\hat{W}}_{c},{\hat{u}}_{c},{\hat{d}}_{w}\right)-H\left(x,\nabla V^{*},u_{c}^{*},d^{*}\right)=H\left(x,{\hat{W}}_{c},{\hat{u}}_{c},{\hat{d}}_{w}\right). \end{array}$$

To effectively approximate the cost function, one needs to adjust the critic NN weight ${\hat{W}}_{c}$ in a manner that minimizes the Hamiltonian error $e_{c}$. To this end, it is common practice to train the critic NN by minimizing the squared residual error $E_{c}$, where $E_{c}=e_{c}^{T}e_{c}/2$. The traditional weight updating laws of critic NN based on gradient descent method can only minimize the squared error, but cannot provide any guarantee for the stability of the resulting system during the learning phase.

However, in practice, the stability is one fundamental requirement of system, and a prerequisite for achieving other higher performance. Thus, not just for minimizing the residual error, but also to guarantee the system stability and eliminate the need for an initial stabilizing control, a weight updating law is developed for the critic NN as follows:

(49) $$\begin{array}{cc} {\dot{\hat{W}}}_{c}= & -\alpha \frac{\phi }{{\left(\phi^{T}\phi +1\right)}^{2}}e_{c}+\frac{\alpha }{4}\nabla \sigma_{c}D{\left(\nabla \sigma_{c}\right)}^{T}{\hat{W}}_{c}\frac{\phi_{1}^{T}}{\phi_{s}}{\hat{W}}_{c}-\alpha \left(F_{2}-F_{1}\phi_{1}^{T}\right){\hat{W}}_{c}\\ \; & +\frac{\beta }{2}\Sigma \left(x,{\hat{u}}_{c},{\hat{d}}_{w}\right)\nabla \sigma_{c}D\nabla J_{a}, \end{array}$$

where α and β are the positive updating ratios, $\phi =\nabla \sigma_{c}\left(f\left(x\right)-D{\left(\nabla \sigma_{c}\right)}^{T}{\hat{W}}_{c}/2\right)$, $\phi_{1}=\phi /\left(\phi^{T}\phi +1\right)$, $\phi_{s}=\phi^{T}\phi +1$, $F_{1}$ and $F_{2}$ represent design parameter matrices with suitable dimensions, $J_{a}\left(x\right)$ is a Lyapunov function candidate provided in Assumption 4, and the index operator $\Sigma (x,{\hat{u}}_{c},{\hat{d}}_{w})$ is given by

(50) $$\begin{array}{c} \Sigma \left(x,{\hat{u}}_{c},{\hat{d}}_{w}\right)=\left\{\begin{array}{ccc} \; & 0, & \;\;\;\mathrm{if}\;\;{\dot{J}}_{a}\left(x\right)={\left(\nabla J_{a}\right)}^{T}(f\left(x\right)+g\left(x\right){\hat{u}}_{c}+k\left(x\right){\hat{d}}_{w})<0\\ \; & 1, & \;\;\;\;\;\;\;\;\;\;\;\;\;\mathrm{otherwise} \end{array}\right. \end{array}$$

with $\nabla J_{a}=\partial J_{a}\left(x\right)/\partial x$.

**Remark** **2.** *Note that in (49), the first term is designed by the normalized gradient descent method for minimizing the residual error. The second term has a well-designed form for ensuring the system’s stability, which is derived from the Lyapunov stability analysis. The last term is an additional adjustment term that works or not depends on the index operator $\Sigma (x,{\hat{u}}_{c},{\hat{d}}_{w})$, which is selected based on the derivative of $J_{a}\left(x\right)$ along the sliding-mode dynamics (30), namely, $\dot{J_{a}}\left(x\right)={\left(\nabla J_{a}\right)}^{T}(f\left(x\right)+g\left(x\right){\hat{u}}_{c}+k\left(x\right){\hat{d}}_{w})$. Once the system dynamics may become unstable, this results in $\dot{J_{a}}\left(x\right)\geq 0$, then $\Sigma (x,{\hat{u}}_{c},{\hat{d}}_{w})=1$ and the last term in (49) is activated. Moreover, based on the negative gradient direction of $\dot{J_{a}}\left(x\right)$, i.e., $-\partial ({\left(\nabla J_{a}\right)}^{T}\left(f\left(x\right)-D\nabla \sigma_{c}^{T}{\hat{W}}_{c}/2\right))/\partial {\hat{W}}_{c}$, the last term is designed to reinforce the training process of the critic NN until the system dynamics become stable. This also eliminates the need for an initial stabilizing control, compared with [35,36,37,39], where the stabilizing control is required for initialization; however, in practical applications, finding an initial stabilizing control is quite challenging.*

**Remark** **3.** *Based on [14,15,16], it is necessary to satisfy the persistence of excitation (PE) requirement for updating the weights of critic NN, which enhances its ability to explore the state space and is indispensable for the weights to converge to their desired ones. To fulfill the PE requirement, a probing noise is injected into the control input [15], which may cause the instability problem during the online learning. As a result, it is important to design the last term in (49) for stabilizing the resulting system, especially when the probing signal is injected.*

**Figure 1:**
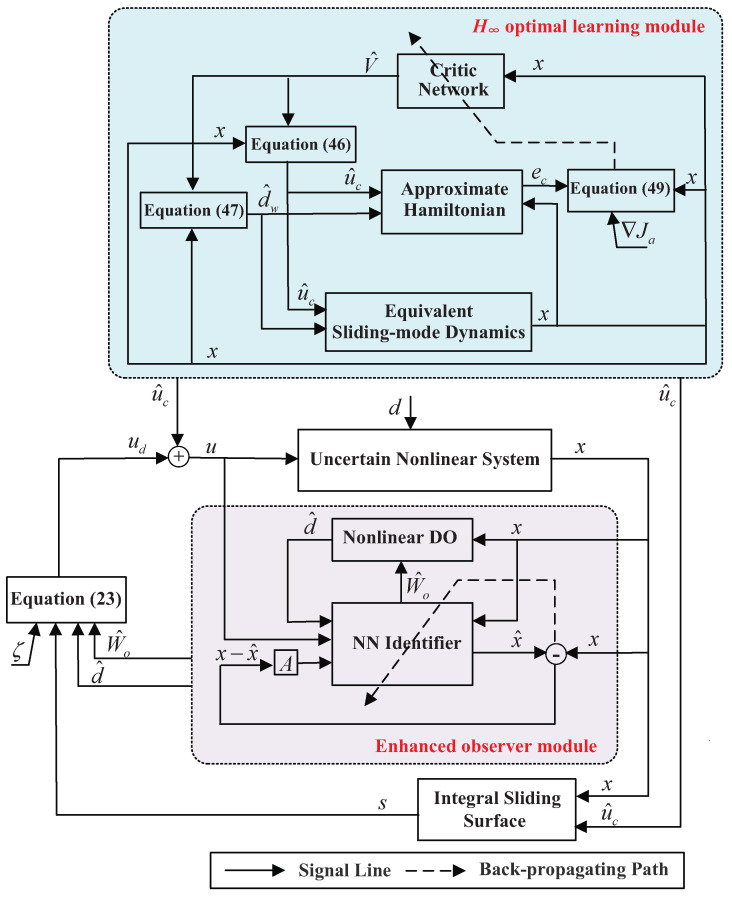
The schematic of the adaptive $H_{\infty }$ SMC scheme.

The schematic structure of the proposed $H_{\infty }$ SMC scheme is illustrated in Figure 1. As shown in Figure 1, this structure consists of two main modules: the $H_{\infty }$ optimal learning module and the enhanced observer module. It should be noted that, based on the deduced sliding-mode dynamics, the learning module can operate independently. However, the original system and the observer module rely on the compound control input *u*, which includes the approximate $H_{\infty }$ optimal control ${\hat{u}}_{c}$ obtained from the learning module. Consequently, it is necessary to first run the learning module to obtain the approximate optimal control ${\hat{u}}_{c}$ during the implementation process.

Considering (43), together with ${\tilde{W}}_{c}=W_{c}-{\hat{W}}_{c}$, (48) is represented as

(51) $$e_{c}=-{\tilde{W}}_{c}^{T}\nabla \sigma_{c}\left(f\left(x\right)-\frac{1}{2}D{\left(\nabla \sigma_{c}\right)}^{T}{\hat{W}}_{c}\right)+\frac{1}{4}{\tilde{W}}_{c}^{T}\nabla \sigma_{c}D{\left(\nabla \sigma_{c}\right)}^{T}{\tilde{W}}_{c}+\varepsilon_{\mathrm{HJI}}.$$

By means of the relation ${\dot{\tilde{W}}}_{c}=-{\dot{\hat{W}}}_{c}$ and incorporating (51) into (49), we obtain

(52) $$\begin{array}{cc} {\dot{\tilde{W}}}_{c}= & -\alpha \frac{\phi_{1}}{\phi_{s}}\left({\tilde{W}}_{c}^{T}\phi -\frac{1}{4}{\tilde{W}}_{c}^{T}\nabla \sigma_{c}D{\left(\nabla \sigma_{c}\right)}^{T}{\tilde{W}}_{c}-\varepsilon_{\mathrm{HJI}}\right)-\frac{\alpha }{4}\nabla \sigma_{c}D{\left(\nabla \sigma_{c}\right)}^{T}{\hat{W}}_{c}\frac{\phi_{1}^{T}}{\phi_{s}}{\hat{W}}_{c}\\ \; & +\alpha \left(F_{2}-F_{1}\phi_{1}^{T}\right){\hat{W}}_{c}-\frac{\beta }{2}\Pi \left(x,{\hat{u}}_{c},{\hat{d}}_{w}\right)\nabla \sigma_{c}D\nabla J_{a}. \end{array}$$

Next, the main stability theorem is presented, but before that, one basic common assumption for the critic NN is introduced [16], and the other assumption for the sliding-mode dynamics is also needed, which has been used in [34,38].

**Assumption** **3.** *For the critic NN, there exist known positive constants $\sigma_{\mathrm{cM}}$, $\sigma_{\mathrm{dM}}$, $\varepsilon_{\mathrm{cM}}$, $\varepsilon_{\mathrm{dM}}$ and $W_{\mathrm{cM}}$ such that $∥\sigma_{c}\left(x\right)∥\leq \sigma_{\mathrm{cM}}$, $∥\nabla \sigma_{c}∥\leq \sigma_{\mathrm{dM}}$, $∥\varepsilon_{c}\left(x\right)∥\leq \varepsilon_{\mathrm{cM}}$, $∥\nabla \varepsilon_{c}∥\leq \varepsilon_{\mathrm{dM}}$ and $∥W_{c}∥\leq W_{\mathrm{cM}}$, respectively. Moreover, the approximation error $\varepsilon_{\mathrm{HJI}}$ is bounded above by $\varepsilon_{H}>0$, namely, $∥\varepsilon_{\mathrm{HJI}}∥\leq \varepsilon_{H}$.*

**Assumption** **4.** Considering the sliding-mode dynamics (30) with the optimal control pair $\left(u_{c}^{*},d^{*}\right)$ in (36) and (37), let $J_{a}\left(x\right)$ be a smooth, radially unbounded and positive definite Lyapunov candidate that satisfies $\dot{J_{a}}\left(x\right)={\left(\nabla J_{a}\right)}^{T}\left(f\left(x\right)+g\left(x\right)u_{c}^{*}+k\left(x\right)d^{*}\right)<0$. Moreover, it is assumed that a positive definite matrix Ψ(x) makes ${\left(\nabla J^{*}\right)}^{T}\Psi \left(x\right)\nabla J_{a}=x^{T}Qx+u_{c}^{*T}Ru_{c}^{*}-\gamma^{2}d^{*T}d^{*}$ hold. Then, one can derive

(53) $${\left(\nabla J_{a}\right)}^{T}\left(f\left(x\right)+g\left(x\right)u_{c}^{*}+k\left(x\right)d^{*}\right)=-{\left(\nabla J_{a}\right)}^{T}\Psi \left(x\right)\nabla J_{a}.$$

**Remark** **4.** *Note that the plausibility of Assumption 4 depends on the boundedness of optimal sliding-mode dynamics, which is usually assumed to be bounded by a function of system state x. For more details, refer to [34,38]. Furthermore, it is impossible to solve (53) directly for getting the form of $J_{a}\left(x\right)$. Based on [34], one can obtain $J_{a}\left(x\right)$ by selecting an appropriate form, such as a quadratic polynomial.*

**Theorem** **2.** Considering the sliding-mode dynamics (30) and its associated cost function (32), the control input and disturbance policy are designed by (46) and (47), respectively, along with the critic weight updating law as given by (49). Then, both the sliding-mode state x and the weight estimation error ${\tilde{W}}_{c}$ are ensured to be UUB. Furthermore, the obtained control input ${\hat{u}}_{c}$ can be proven to converge to a neighborhood of the optimum control $u_{c}^{*}$ with a small adjustable bound.

**Proof.**  Consider the following Lyapunov function candidate

$$L=\frac{1}{2}{\tilde{W}}_{c}^{T}\alpha^{-1}{\tilde{W}}_{c}+\beta_{1}J_{a}\left(x\right),$$

where $\beta_{1}=\beta /\alpha >0$. By calculating the time derivative of *L* along the sliding-mode dynamics (30), we have

(54) $$\dot{L}={\tilde{W}}_{c}^{T}{\dot{\tilde{W}}}_{c}+\beta_{1}{\left(\nabla J_{a}\right)}^{T}(f\left(x\right)+g\left(x\right){\hat{u}}_{c}+k\left(x\right){\hat{d}}_{w}).$$

Substituting (52) into (54) and making some adjustments, one can get

(55) $$\begin{array}{cc} \dot{L}= & -{\tilde{W}}_{c}^{T}\phi_{1}\phi_{1}^{T}{\tilde{W}}_{c}+\beta_{1}{\left(\nabla J_{a}\right)}^{T}(f\left(x\right)+g\left(x\right){\hat{u}}_{c}+k\left(x\right){\hat{d}}_{w})+\frac{1}{4}{\tilde{W}}_{c}^{T}\nabla \sigma_{c}D{\left(\nabla \sigma_{c}\right)}^{T}W_{c}\frac{\phi_{1}^{T}}{\phi_{s}}{\tilde{W}}_{c}\\ \; & -\frac{1}{4}{\tilde{W}}_{c}^{T}\nabla \sigma_{c}D{\left(\nabla \sigma_{c}\right)}^{T}W_{c}\frac{\phi_{1}^{T}}{\phi_{s}}W_{c}-\frac{1}{4}{\tilde{W}}_{c}^{T}\nabla \sigma_{c}D{\left(\nabla \sigma_{c}\right)}^{T}{\tilde{W}}_{c}\frac{\phi_{1}^{T}}{\phi_{s}}W_{c}\\ \; & +{\tilde{W}}_{c}^{T}\frac{\phi_{1}^{T}}{\phi_{s}}\varepsilon_{\mathrm{HJI}}-\frac{\beta }{2}\Sigma \left(x,{\hat{u}}_{c},{\hat{d}}_{w}\right){\tilde{W}}_{c}^{T}\nabla \sigma_{c}D\nabla J_{a}\\ \; & +{\tilde{W}}_{c}^{T}F_{2}{\hat{W}}_{c}-{\tilde{W}}_{c}^{T}F_{1}\phi_{1}^{T}{\hat{W}}_{c}. \end{array}$$

Using ${\hat{W}}_{c}=W_{c}-{\tilde{W}}_{c}$, the last two terms in (55) become

(56) $${\tilde{W}}_{c}^{T}F_{2}{\hat{W}}_{c}-{\tilde{W}}_{c}^{T}F_{1}\phi_{1}^{T}{\hat{W}}_{c}={\tilde{W}}_{c}^{T}F_{2}W_{c}-{\tilde{W}}_{c}^{T}F_{2}{\tilde{W}}_{c}-{\tilde{W}}_{c}^{T}F_{1}\phi_{1}^{T}W_{c}+{\tilde{W}}_{c}^{T}F_{1}\phi_{1}^{T}{\tilde{W}}_{c}.$$

Defining $\Upsilon ={\left[{\tilde{W}}_{c}^{T}\phi_{1},{\tilde{W}}_{c}^{T}\right]}^{T}$, and substituting (55) into (56), it can be rewritten as

(57) $$\begin{array}{cc} \dot{L}= & -\Upsilon^{T}M\Upsilon +\Upsilon^{T}\delta +\beta_{1}{\left(\nabla J_{a}\right)}^{T}(f\left(x\right)+g\left(x\right){\hat{u}}_{c}+k\left(x\right){\hat{d}}_{w})\\ \; & -\frac{\beta }{2}\Sigma \left(x,{\hat{u}}_{c},{\hat{d}}_{w}\right){\tilde{W}}_{c}^{T}\nabla \sigma_{c}D\nabla J_{a}, \end{array}$$

where

$$\begin{array}{cc} M & =\left[\begin{array}{cc} I & -\frac{\nabla \sigma_{c}D{\left(\nabla \sigma_{c}\right)}^{T}W_{c}}{4\phi_{s}}-\frac{F_{1}}{2}\\ -\frac{\nabla \sigma_{c}D{\left(\nabla \sigma_{c}\right)}^{T}W_{c}}{4\phi_{s}}-\frac{F_{1}}{2} & F_{2} \end{array}\right],\\ \delta & =\left[\begin{array}{c} \frac{1}{\phi_{s}}\varepsilon_{\mathrm{HJI}}\\ -\frac{\nabla \sigma_{c}D{\left(\nabla \sigma_{c}\right)}^{T}W_{c}}{4\phi_{s}}+F_{2}W_{c}-F_{1}\phi_{1}^{T}W_{c} \end{array}\right]. \end{array}$$

With Assumption 3 in mind, and recalling the boundedness of $\phi_{1}$ and *D*, in particular $∥\phi_{1}∥<1$ and $∥D∥\leq D_{M}$, we can infer that there exists a positive constant $\delta_{M}$ in the sense that $∥\delta ∥\leq \delta_{M}$. For guaranteeing M>0, the appropriate parameters $F_{1}$ and $F_{2}$ need to be selected in design. Then, one can upper bound $\dot{L}$ as follows:

(58) $$\begin{array}{cc} \dot{L}\leq & -\lambda_{\min}{\left(M\right)∥\Upsilon ∥}^{2}+\delta_{M}∥\Upsilon ∥+\beta_{1}{\left(\nabla J_{a}\right)}^{T}(f\left(x\right)+g\left(x\right){\hat{u}}_{c}+k\left(x\right){\hat{d}}_{w})\\ \; & -\frac{\beta_{1}}{2}\Sigma \left(x,{\hat{u}}_{c},{\hat{d}}_{w}\right){\tilde{W}}_{c}^{T}\nabla \sigma_{c}D\nabla J_{a} \end{array}$$

with $\lambda_{\min}\left(M\right)$ being the minimum eigenvalue of *M*.

According to (50), there are two cases to consider: $\Sigma (x,{\hat{u}}_{c},{\hat{d}}_{w})=0$ and $\Sigma (x,{\hat{u}}_{c},{\hat{d}}_{w})=1$ for (58) in the following analysis.

Case *1*: For $\Sigma (x,{\hat{u}}_{c},{\hat{d}}_{w})=0$, it follows from (50) that ${\dot{J}}_{a}\left(x\right)<0$, i.e., ${\left(\nabla J_{a}\right)}^{T}\dot{x}<0$, which, together with the PE condition, can ensure that there exists a positive constant ϱ such that $∥\dot{Z}∥>\varrho$. This implies that ${\left(\nabla J_{a}\right)}^{T}\dot{x}<-\varrho ∥\nabla J_{a}∥<0$. Then, (58) becomes

(59) $$\begin{array}{cc} \dot{L} & \leq \beta_{1}{\left(\nabla J_{a}\right)}^{T}\dot{x}-\lambda_{\min}\left(M\right){∥\Upsilon ∥}^{2}+\delta_{M}∥\Upsilon ∥\\ \; & <-\beta_{1}\varrho ∥\nabla J_{a}∥-\lambda_{\min}\left(M\right){\left(∥\Upsilon ∥-\frac{\delta_{M}}{2\lambda_{\min}\left(M\right)}\right)}^{2}+\frac{\delta_{M}^{2}}{4\lambda_{\min}\left(M\right)}. \end{array}$$

Focus on (59), only if the following inequalities:

$$∥\nabla J_{a}∥>\frac{\delta_{M}^{2}}{4\lambda_{\min}\left(M\right)\varrho }≜A_{1}$$

or

$$∥\Upsilon ∥>\frac{\delta_{M}^{2}}{\lambda_{\min}\left(M\right)}$$

hold, then $\dot{L}<0$. Moreover, based on the relation $∥\Upsilon ∥\leq \sqrt{∥\phi_{1}{∥}^{2}+1}∥{\tilde{W}}_{c}∥$ with $∥\phi_{1}∥<1$, we can derive

$$∥{\tilde{W}}_{c}∥>\frac{\delta_{M}^{2}}{\sqrt{2}\lambda_{\min}\left(M\right)}≜B_{1}.$$

Case *2*: For $\Sigma (x,{\hat{u}}_{c},{\hat{d}}_{w})=1$, in light of (41) and (42), by adding and subtracting $\beta_{1}{\left(\nabla J_{a}\right)}^{T}D\nabla \varepsilon_{c}/2$ into (58), we can derive

(60) $$\begin{array}{cc} \dot{L}\leq & -\lambda_{\min}\left(M\right){\left(∥\Upsilon ∥-\frac{\delta_{M}}{2\lambda_{\min}\left(M\right)}\right)}^{2}+\frac{\delta_{M}^{2}}{4\lambda_{\min}\left(M\right)}+\beta_{1}{\left(\nabla J_{a}\right)}^{T}(f\left(x\right)+g\left(x\right)u_{c}^{*}\\ \; & +k\left(x\right)d^{*})+\frac{\beta_{1}}{2}{\left(\nabla J_{a}\right)}^{T}D\nabla \varepsilon_{c}. \end{array}$$

Then, using (53) in Assumption 4, and recalling the boundedness of *D* and $\nabla \varepsilon_{c}$, (60) is upper bounded as

(61) $$\dot{L}\leq -\lambda_{\min}\left(M\right){\left(∥\Upsilon ∥-\frac{\delta_{M}}{2\lambda_{\min}\left(M\right)}\right)}^{2}-\frac{\beta_{1}}{2}\lambda_{\min}\left(\Psi \right){∥\nabla J_{a}∥}^{2}+\Phi ,$$

where $\Phi =\delta_{M}^{2}/\left(4\lambda_{\min}\left(M\right)\right)+\beta_{1}D_{M}^{2}\varepsilon_{\mathrm{dM}}^{2}/\left(8\lambda_{\min}\left(\Psi \right)\right)$, $\lambda_{\min}\left(\Psi \right)$ denotes the minimum eigenvalue of Ψ(x). Hence, provided the following inequalities:

$$∥\nabla J_{a}∥>\sqrt{\frac{2\Phi }{\beta_{1}\lambda_{\min}\left(\Psi \right)}}≜A_{2}$$

or

$$∥\Upsilon ∥>\sqrt{\frac{\Phi }{\lambda_{\min}\left(M\right)}}+\frac{\delta_{M}}{2\lambda_{\min}\left(M\right)}$$

hold, one has $\dot{L}<0$. Further, by the relation $∥\Upsilon ∥\leq \sqrt{2}∥{\tilde{W}}_{c}∥$, we have

$$∥{\tilde{W}}_{c}∥>\sqrt{\frac{\Phi }{2\lambda_{\min}\left(M\right)}}+\frac{\delta_{M}}{2\sqrt{2}\lambda_{\min}\left(M\right)}≜B_{2}.$$

To sum up, for both Case *1* and Case *2*, with proper parameters $F_{1}$ and $F_{2}$ satisfying M>0, the inequality $∥\nabla J_{a}∥\geq \max\left\{A_{1},A_{2}\right\}=\bar{A}$ or $∥{\tilde{W}}_{c}∥\geq \max\left\{B_{1},B_{2}\right\}=\bar{B}$ holds, then, we have $\dot{L}<0$. From the Lyapunov extension theorem [16], it is found that $∥\nabla J_{a}∥$ and $∥{\tilde{W}}_{c}∥$ are bounded by $\bar{A}$ and $\bar{B}$, respectively. Based on Assumption 4, the Lyapunov candidate $J_{a}\left(x\right)$ is radially unbounded, which implies that the boundedness of $∥\nabla J_{a}∥$ leads to the boundedness of the system state ∥x∥. In particular, ∥x∥ is bounded by ${\bar{A}}_{x}=\max\left\{A_{1x},A_{2x}\right\}$, where $A_{1x}$ and $A_{2x}$ are determined by $A_{1}$ and $A_{2}$, respectively. So far, we can conclude that both *x* and ${\tilde{W}}_{c}$ are guaranteed to be UUB.

Next, we will prove ${\hat{u}}_{c}$ converges to a small neighborhood of $u_{c}^{*}$ with an adjustable bound, i.e., $∥{\hat{u}}_{c}-u_{c}^{*}∥\leq \epsilon_{u}$. Considering (41) and (46), we have

$${\hat{u}}_{c}-u_{c}^{*}=-\frac{1}{2}R^{-1}g^{T}\left(x\right)({\left(\nabla \sigma_{c}\right)}^{T}{\tilde{W}}_{c}+\nabla \varepsilon_{c}).$$

Noticing that ${\tilde{W}}_{c}$ is UUB together with the associated bound $\bar{B}=\max\left\{B_{1},B_{2}\right\}$, and invoking $∥g\left(x\right)∥\leq g_{M}$, $∥\nabla \sigma_{c}∥\leq \sigma_{\mathrm{dM}}$, $∥\nabla \varepsilon_{c}∥\leq \varepsilon_{\mathrm{dM}}$ and boundedness of *R*, it follows that

(62) $$\begin{array}{cc} ∥{\hat{u}}_{c}-u_{c}^{*}∥ & \leq \frac{1}{2}\lambda_{\max}\left(R^{-1}\right)g_{M}\left(\sigma_{\mathrm{dM}}\bar{B}+\varepsilon_{\mathrm{dM}}\right)≜\epsilon_{u}. \end{array}$$

 □

**Remark** **5.** *From the expression of $B_{1}$ and $B_{2}$, it is seen that $\bar{B}$ can be kept small with $\lambda_{\min}\left(M\right)$ being larger enough. In view of (57), we can enlarge the value of $\lambda_{\min}\left(M\right)$ by adjusting the corresponding design parameters $F_{1}$ and $F_{2}$. Moreover, we can make the approximate error $\varepsilon_{c}$ and its upper bound $\varepsilon_{\mathrm{dM}}$ sufficiently small when the neuron number $l_{c}$ is large enough. Therefore, we can make the convergence errors $\epsilon_{u}$ in (62) as small as possible in the design.*

## 5. Simulation Results

To validate the effectiveness of the proposed $H_{\infty }$ optimal SMC scheme, two simulation examples are provided. The first example focuses on a single-link robot arm, while the second example deals with a power system.

### 5.1. Single-Link Robot Arm

Considering a nonlinear single-link robot arm [23] and its dynamics given by

(63) $$J\ddot{\theta }=-MgL\sin\left(\theta \right)-D\dot{\theta }+u+w,$$

where θ is the joint rotation angle of robot arm in radians, *u* refers to the control torque applied to the joint in Nm, and *w* denotes the lumped uncertain term. Select the system parameters as follows: the arm length L=0.5m, the payload mass M=1kg, the local gravity acceleration $g=9.81\;m/s^{2}$. the rotational inertia $J=1\;\mathrm{kg}\cdot m^{2}$ and the viscous friction D=2Nm·s/rad. With the system states defined as $x_{1}=\theta$ and $x_{2}=\dot{\theta }$, and considering the presence of exogenous disturbances, then the dynamics (63) in state-space form can be represented as

(64) $$\left[\begin{array}{c} {\dot{x}}_{1}\\ {\dot{x}}_{2} \end{array}\right]=\left[\begin{array}{c} x_{2}\\ -4.905\sin\left(x_{1}\right)-2x_{2} \end{array}\right]+\left[\begin{array}{c} 0\\ 1 \end{array}\right]\left(u+w\right)+d,$$

where *d* represents the unknown disturbances. Moreover, it is assumed that the initial state is set as $x_{0}={\left[1,-0.5\right]}^{T}$, the lumped uncertainty term is $w\left(x,u\right)=x_{2}\sin\left(x_{1}\right)+0.1\sin\left(x_{1}\right)u$, and the disturbance term is chosen as $d={\left[0.5e^{-t}\sin\left(t\right),0.5\sin\left(t\right)\right]}^{T}$ in the simulation.

The enhanced observer system, consisting of an NN identifier and a nonlinear DO, can be designed as shown in (6), where the identifier NN is selected as a three-layered feedforward NN with one hidden layer containing six neurons, and the hyperbolic activation function tanh(·) is utilized. The updating ratios are set as $\eta_{1}=30$ and $\eta_{2}=2.5$, while the weights ${\hat{W}}_{o}$ and ${\hat{V}}_{o}$ are initialized with random values chosen from the interval [−0.1,0.1]. The initial observer state is set as ${\hat{x}}_{0}={\left[0.5,0\right]}^{T}$. Moreover, based on Lemma 1, select the Hurwitz matrix A=[−15,0;0,−15], $p\left(x\right)=\left[10x_{1};10x_{2}\right]$ and l(x)=[10,0;0,10] to ensure that the inequality (10) holds. The integral sliding surface function is determined by (21), together with $G\left(x\right)=g^{+}\left(x\right)=\left[0,1\right]$ and $S_{0}\left(x\right)=x_{2}$. Accordingly, the discontinuous SMC $u_{d}$ is given by (23) and (24). For the propose of eliminating the chattering phenomenon, an arctangent function atan(s/ϵ) with a small positive scalar ϵ=0.005 is employed to replace the sign function sgn(s) in (23).

By considering the SMC law $u_{d}$, the sliding-mode dynamics can be obtained as

(65) $$\dot{x}=f\left(x\right)+g\left(x\right)u_{c}+k\left(x\right)d,$$

where $k\left(x\right)=I-g\left(x\right)g^{+}\left(x\right)=\left[1,0;0,0\right]$. We choose the associated cost function as the form of (32), together with Q=diag(1,1), R=1 and γ=1.5. For the critic NN, the activation function is chosen as $\sigma_{c}\left(x\right)={\left[x_{1}^{2},x_{1}x_{2},x_{2}^{2},x_{1}^{3}x_{2},x_{1}^{2}x_{2}^{2},x_{1}x_{2}^{3}\right]}^{T}$, which results in ${\hat{W}}_{c}={\left[{\hat{W}}_{c1},{\hat{W}}_{c2},\ldots ,{\hat{W}}_{c6}\right]}^{T}$. Select the updating ratios α=1, β=0.5, the design parameters $F_{1}=F_{2}=10I$, $l_{c}=6$ and $J_{a}\left(x\right)$ as a quadratic polynomial. Furthermore, the weight vector ${\hat{W}}_{c}$ is initialized to zero, which leads to the initial control input of zero. Noticing that the zero initial control cannot make the system (65) stable, it is thus clear that no initial stabilizing control strategy is necessary when implementing the proposed algorithm.

**Figure 2:**
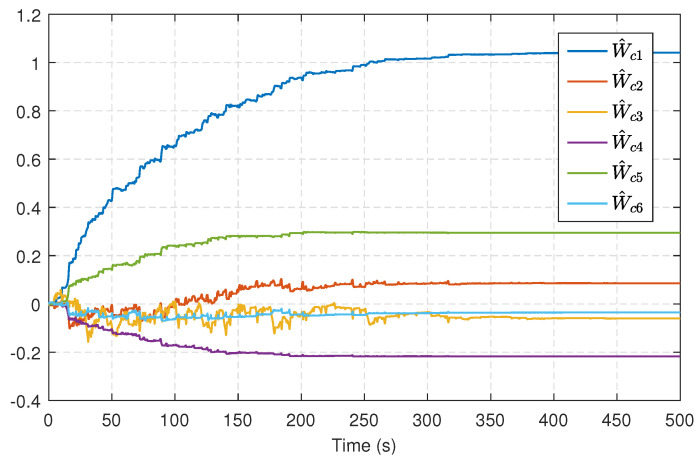
Trajectories of the critic NN weights.

**Figure 3:**
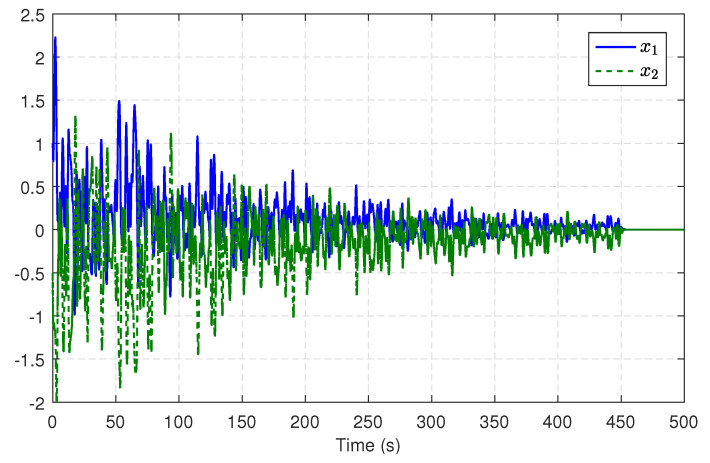
Trajectories of system states in the learning.

During the learning process, a damped decreasing probing noise is injected into the control input for satisfying PE condition. This noise comprises sinusoids of diverse frequencies and is applied for the first 450 s. Figure 2 shows the trajectories of the critic weights, which eventually converge to ${\hat{W}}_{c}={\left[1.0420,0.0856,-0.0603,-0.2174,0.2948,-0.0358\right]}^{T}$. Figure 3 describes the trajectories of system states in the learning. From Figure 3, one can see that without an initial stabilizing control, the system states stay at or near zero after the probing noise is removed, which indicates that ${\hat{u}}_{c}$ generated by the learning module can effectively stabilize the system. With the converged weights, the approximate $H_{\infty }$ optimal control ${\hat{u}}_{c}$ can be calculated by (46).

**Figure 4:**
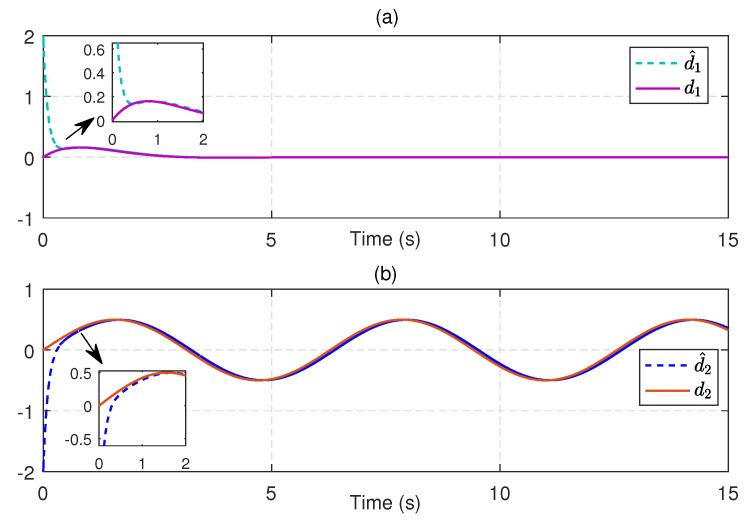
(**a**) Real disturbance $d_{1}$ and its estimation ${\hat{d}}_{1}$, (**b**) Real disturbance $d_{2}$ and its estimation ${\hat{d}}_{2}$.

**Figure 5:**
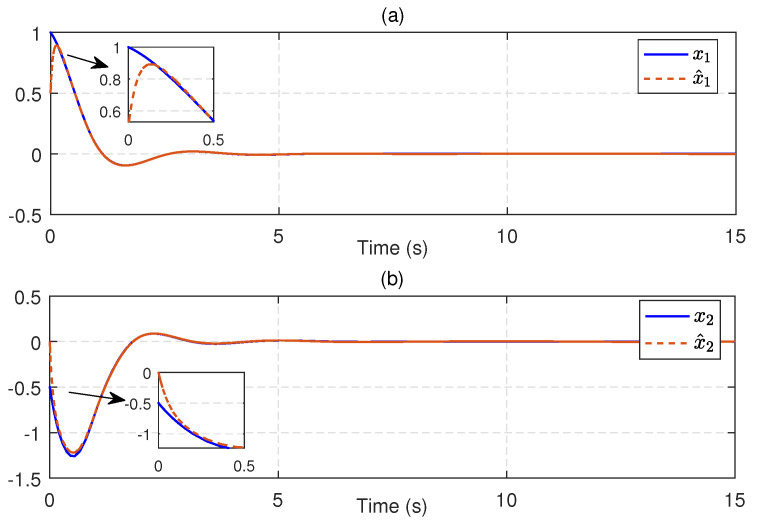
(**a**) Real state $x_{1}$ and identified state ${\hat{x}}_{1}$, (**b**) Real state $x_{2}$ and identified state ${\hat{x}}_{2}$.

**Figure 6:**
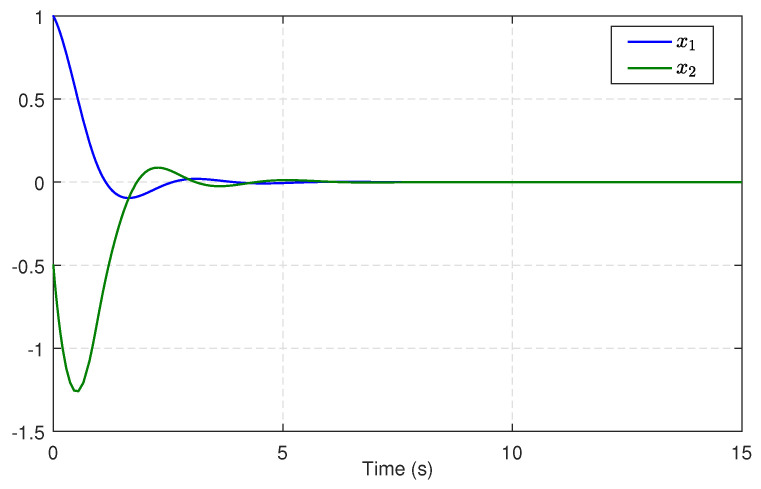
State trajectories of the robotic arm.

**Figure 7:**
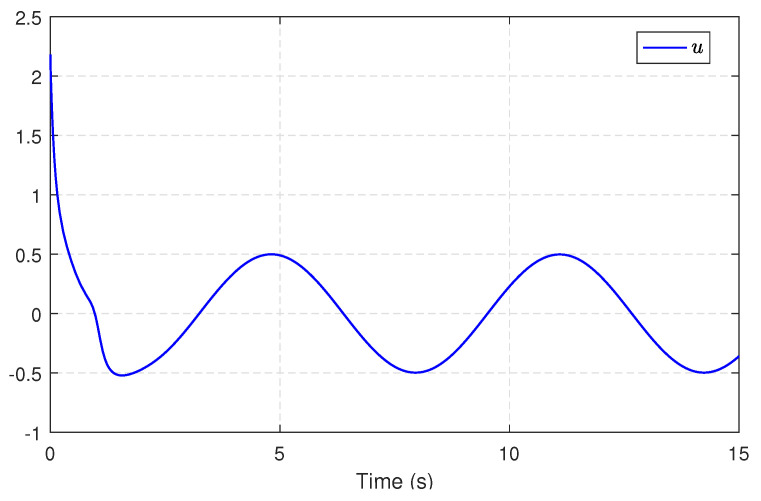
The compound control *u*.

**Figure 8:**
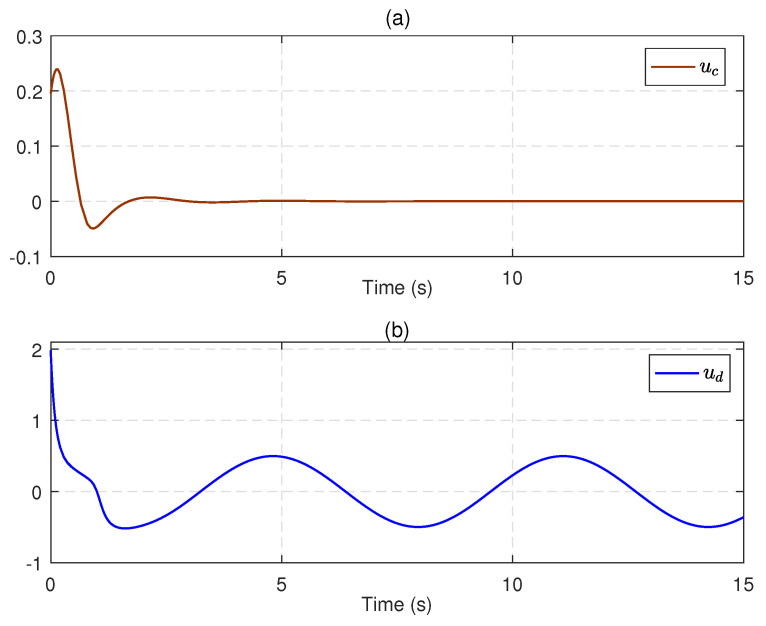
(**a**) The $H_{\infty }$ optimal control ${\hat{u}}_{c}$. (**b**) The SMC law $u_{d}$.

Next, we substitute ${\hat{u}}_{c}$ into (21) to obtain an available sliding surface. Subsequently, integrating with the enhanced observer system, the SMC law $u_{d}$ is implemented by using (23) and (24) with the reliable estimations of uncertainties and disturbances. Figure 4 depicts the estimates of disturbances $d_{1}=0.5e^{-t}\sin\left(t\right)$ and $d_{2}=0.5\sin\left(t\right)$, along with small estimation errors. Figure 5 presents the identifications of system states using the identifier NN. It can be observed that the identified states rapidly track the real states, illustrating the effectiveness and efficiency of the identifier NN. Note that the valid estimations $\hat{d}$ and ${\hat{W}}_{o}$ are used to design the SMC law $u_{d}$, which helps to reduce the sliding-mode gain and alleviate the chattering phenomenon. Figure 6 displays the state trajectories of the robot arm under the compound $H_{\infty }$ sliding-mode control $u=u_{d}+{\hat{u}}_{c}$. Figure 7 depicts the compound control *u*, while the $H_{\infty }$ control ${\hat{u}}_{c}$ and the SMC law $u_{d}$ are given in Figure 8. These results presented in Figure 6, Figure 7 and Figure 8 confirm that the compound control *u* successfully renders the robot arm system stable and exhibits satisfactory performance against both system uncertainties and external disturbances.

### 5.2. Power Plant System

To further validate the effectivity of the proposed scheme, we consider an electric power system comprised of a gas turbine generator, a system load, and an automatic generation control [34]. To model this system, the incremental frequency deviation $\Delta f_{G}$, the generator output power variation $\Delta P_{m}$, and the valve position change of the governor Δv are taken into consideration. The control input is represented by the speed change $\Delta P_{c}$ in position deviation. By defining the state vector $x={\left[\Delta v,\Delta P_{m},\Delta f_{G}\right]}^{T}\in R^{3}$, we can express the reduced power system model in state-space form as

(66) $$\dot{x}=\left[\begin{array}{ccc} -\frac{1}{T_{g}} & 0 & \frac{1}{R_{g}T_{g}}\\ \frac{K_{t}}{T_{t}} & -\frac{1}{T_{t}} & 0\\ 0 & \frac{K_{p}}{T_{p}} & -\frac{1}{T_{p}} \end{array}\right]x+\left[\begin{array}{c} \frac{1}{T_{g}}\\ 0\\ 0 \end{array}\right]\left(u+\vartheta \right)+d$$

where $g\left(x\right)={\left[1/T_{g},0,0\right]}^{T}$, ϑ represents the modeling uncertainty, and *d* stands for the exterior disturbances. Assume that the uncertain term is $\vartheta =x_{2}\sin\left(x_{1}\right)$, and the disturbance term is defined as $d\left(t\right)={\left[\sin\left(2\pi t\right)e^{-t},0,0.2\sin^{2}\left(t\right)e^{-t}\right]}^{T}$ in the simulation. Let the regulation constant $R_{g}$ = 2.5 Hz/MW, the turbine gain constant $K_{t}$ = 1 s and the generator gain constant $K_{p}$ = 120 Hz/MW. Moreover, the corresponding time constants are set as $T_{g}$ = 0.08 s, $T_{t}$ = 0.1 s and $T_{p}$ = 20 s, respectively.

For estimating the unknown uncertainty and disturbance terms, the enhanced observer system is constructed as (6) with a three-layered feedforward NN containing eight hidden neurons and the Hurwitz matrix A=[−12,0,0;0,−12,0;0,0,−12]. The activation function, the initial weights, and the updating ratios are the same as in Section 5.1. Let $p\left(x\right)={\left[10x_{1},0,10x_{3}\right]}^{T}$, l(x)=[10,0,0;0,0,0;0,0,10], $G\left(x\right)=g^{+}\left(x\right)=\left[0.08,0,0\right]$ and $S_{0}\left(x\right)=0.08x_{1}$. Similarly, an arctangent function atan(s/ϵ) is used for designing the SMC law $u_{d}$ instead of the sign function sgn(s).

**Figure 9:**
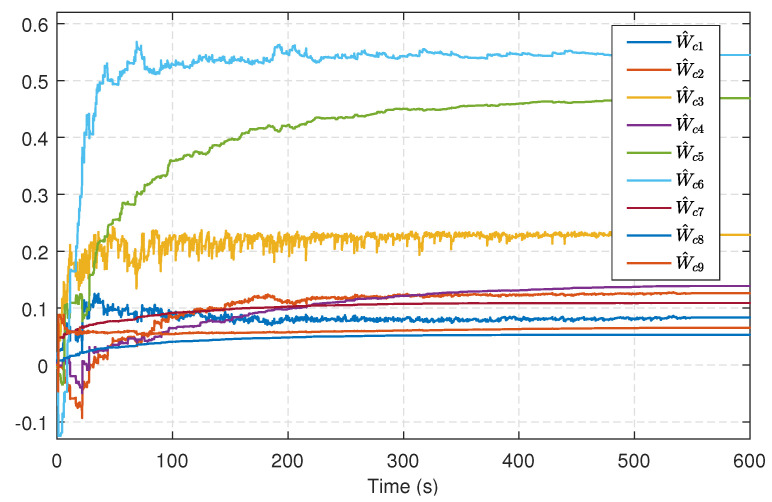
Trajectories of the critic NN weights.

**Figure 10:**
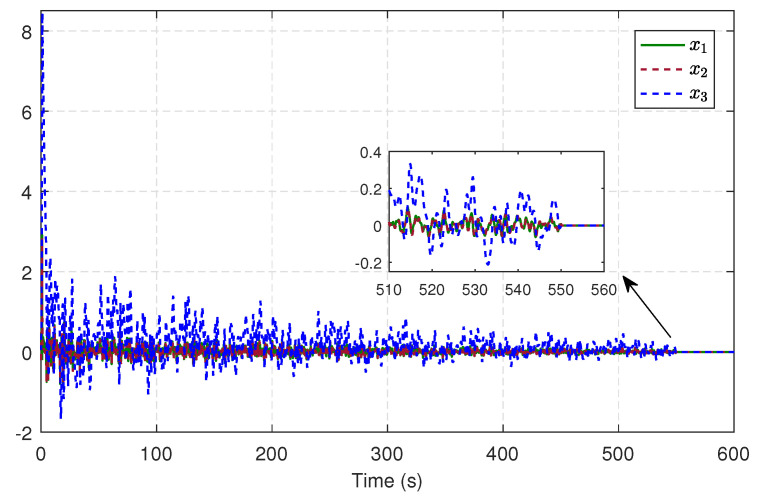
Trajectories of system states in the learning.

Without the matched uncertainties and disturbances, we can derive the sliding-mode dynamics from (66), wherein k(x)=[0,0,0;0,1,0;0,0,1], and the initial state $x_{0}={\left[0.2,-0.2,0.1\right]}^{T}$. Let the associated cost function be of the form (32) along with Q=diag(1,1,1), R=1 and γ=3. The critic NN is designed as (44) and its corresponding parameters are α=15, β=0.5, $\sigma \left(x\right)=[x_{1}^{2},x_{1}x_{2},x_{1}x_{3},x_{2}^{2},x_{2}x_{3},x_{3}^{2},x_{1}^{2}x_{2}x_{3},x_{1}x_{2}^{2}x_{3},x_{1}x_{2}x_{3}^{2}{]}^{T}$ and ${\hat{W}}_{c}={\left[{\hat{W}}_{c1},{\hat{W}}_{c2},\ldots ,{\hat{W}}_{c9}\right]}^{T}$. Similar to Section 5.1, $J_{a}\left(x\right)=x^{T}x/2$, the initial weight vector is set to zero, and a similar probing noise is injected into the control input before 550 s. The evolving trajectories of the critic weights are shown in Figure 9, while the trajectories of system states in the learning are depicted in Figure 10. After 550 s, the critic weights converge to ${\hat{W}}_{c}={\left[0.0830,0.1245,0.2284,0.1616,0.4883,0.5488,0.1154,0.0563,0.0564\right]}^{T}$, then we can derive ${\hat{u}}_{c}$ using (46) with the converged weights.

**Figure 11:**
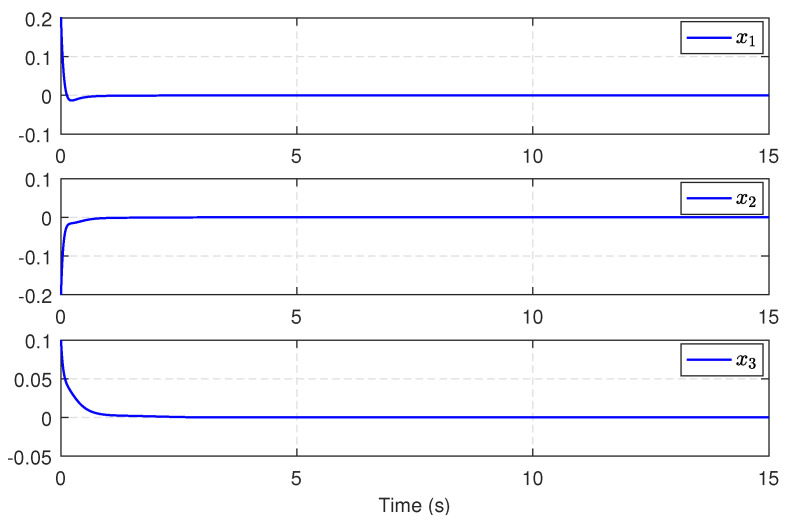
Trajectories of the electric power system.

**Figure 12:**
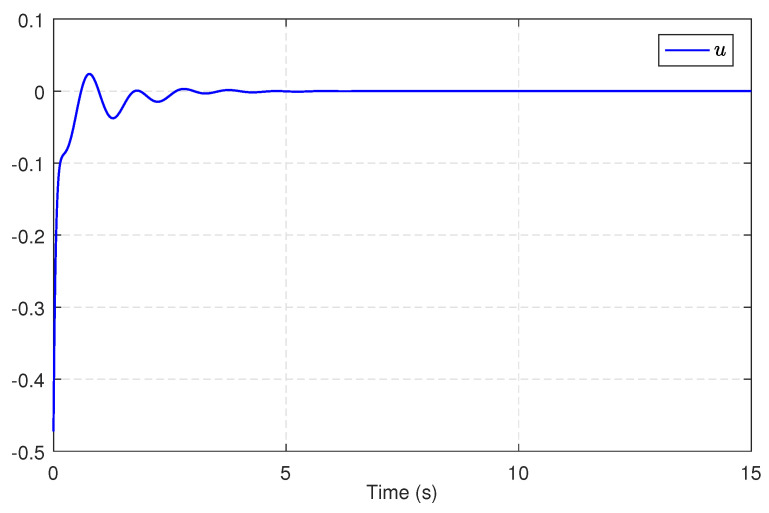
The compound optimal control.

Then, we substitute ${\hat{u}}_{c}$ into the integral sliding surface (21), and we design the SMC law $u_{d}$ by (23) and (24). Consequently, the compound control is constructed as $u=u_{d}+{\hat{u}}_{c}$. After simulation, Figure 11 shows the trajectories of the power system states under this compound control for 15 s. Figure 12 presents the compound control *u*. From Figure 11 and Figure 12, we can conclude that the compound control effectively stabilizes the system states to the equilibrium point, even in the presence of modeling uncertainties and exterior disturbances. These results undeniably demonstrate the viability and efficiency of the proposed approach.

## 6. Conclusions

In this paper, we develop a neural adaptive $H_{\infty }$ sliding-mode control scheme for uncertain nonlinear systems subject to external disturbances. Based on the enhanced observer system composed of the NN identifier and nonlinear DO, an integral SMC is designed for suppressing the influences of the uncertain term and the matched disturbance component, as well as unknown approximation errors, with no prior knowledge of their upper bounds. Meanwhile, on the sliding surface, the remaining unmatched disturbances are attenuated using the $H_{\infty }$ optimal control solved by the single critic network-based ADP algorithm. Furthermore, uniform ultimate boundedness stability of the resultant closed-loop system can be proven by Lyapunov’s method. In addition to the theoretical analysis, two simulation examples are provided to further validate the proposed approach. Recently, the growing interest in saving communication resources or reducing the calculation amount of networked control systems makes the event-triggering mechanism gain more and more attention and undergo rapid development. Hence, how to combine the optimal SMC strategy with the event-triggering mechanism for more complex physical systems, not just for control-affine systems, will be our future research topic.

## Data Availability

The authors can confirm that all relevant data are included in the article.
